# Effects of feeding corn distillers dried grains with solubles diets without or with supplemental enzymes on growth performance of pigs: a meta-analysis

**DOI:** 10.1093/tas/txab029

**Published:** 2021-02-15

**Authors:** Jae-Cheol Jang, Zhikai Zeng, Pedro E Urriola, Gerald C Shurson

**Affiliations:** Department of Animal Science, University of Minnesota, St. Paul, MN 55108, USA

**Keywords:** corn dried distillers grains with solubles, feed enzymes, growing, finishing pigs, growth performance, meta-analysis, nursery pigs

## Abstract

A meta-analysis was conducted to determine the effects of the dietary energy system (net energy or metabolizable energy), oil content of corn distillers dried grains with solubles (cDDGS), diet inclusion levels, and pig age on growth performance of pigs fed cDDGS-based diets. Mean differences of average daily gain (ADG), average daily feed intake (ADFI), and gain:feed (G:F) were calculated and expressed as a percentage change relative to feeding corn–soybean meal (SBM)- and cDDGS-based diets to nursery [body weight (BW) < 25 kg] and growing-finishing (BW > 25 kg) pigs, and to compare the effects of supplementing various types of exogenous enzymes without or with phytase on growth performance. A total of 27 studies with 106 growth performance observations were included in the cDDGS dataset, and 34 studies with 84 observations for enzyme responses in cDDGS diets were used in the enzyme dataset. Approximately, 64.7% of the observations showed no change, and 26.7% of observations showed a reduction in ADG, ADFI, and G:F when feeding cDDGS-based diets to the nursery and growing-finishing pigs compared with feeding corn–SBM-based diets. Furthermore, feeding cDDGS diets resulted in decreased (*P* < 0.01) mean difference of ADG (–4.27%) and G:F (–1.99%) for nursery pigs, and decreased (*P* < 0.01) mean difference of ADG (–1.68%) and G:F (–1.06%) for growing–finishing pigs. Every percentage unit increase in the inclusion level of cDDGS in growing–finishing pig diet was associated with a decrease (*P* < 0.01) in ADG (–0.10%) and ADFI (–0.09%). Feeding high oil (≥10% ether extract) cDDGS-based diets to pigs resulted in a 2.96% reduction in ADFI whereas feeding reduced-oil (<10% ether extract) cDDGS-based diets reduced G:F by 1.56% compared with pigs fed corn–SBM-based diets. Supplementation of exogenous enzymes improved (*P* < 0.05) the mean difference of ADG and G:F by 1.94% and 2.65%, respectively, in corn–SBM-based diets, and by 2.67% and 1.87%, respectively, in cDDGS diets. Supplementation of exogenous protease, enzyme cocktail, or xylanase improved (*P* < 0.05) ADG by 7.29%, 2.64%, and 2.48% in pigs fed corn–SBM-based diets, respectively. There were no differences between the dietary addition of single enzymes and enzyme combinations for any growth performance parameters in corn–SBM- or cDDGS-based diets. In conclusion, feeding cDDGS-based diets slightly reduces the growth performance of nursery and growing–finishing pigs, but supplementation of xylanase or enzyme cocktail can improve G:F of pigs fed cDDGS-based diets.

## INTRODUCTION

Corn distillers dried grains with solubles (cDDGS) is used primarily as an energy source in swine diets because it contains approximately the same amount of metabolizable energy (ME) as corn (NRC, 2012). Therefore, the dietary addition of cDDGS results in replacing a portion of the corn, and a lesser amount of soybean meal and inorganic phosphorus. In a review of over 20 published studies up to 2008, results showed that adding up to 30% cDDGS to the nursery and growing–finishing diets had no effect on growth performance (Stein and Shurson, 2009).

However, beginning in 2005, some U.S. ethanol production facilities began separating some of the corn oil from thin stillage before producing reduced oil cDDGS (Shurson, 2017). Currently, approximately 98% of U.S. ethanol facilities are removing varying proportions of corn oil from thin stillage which has resulted in a wide range of corn oil (4% to 12% ether extract; EE) content among cDDGS sources. This reduction and variability in EE content of cDDGS implied that ME may be reduced, more variable among sources, and negatively affect the growth performance of pigs. Graham et al. (2014a) reported that increasing dietary inclusion rates of medium oil cDDGS (7.63% EE) decreased ADG, gain:feed (G:F), and final body weight (BW) compared with pigs fed corn–soybean meal (SBM)-based diets. However, Kerr et al. (2013) reported that EE content of cDDGS is a poor single predictor of ME content for swine. Furthermore, Wu et al. (2016a) found that pigs fed diets containing 40% reduced oil cDDGS containing variable EE (5.9%, 9.9%, or 14.2% EE) but similar predicted ME, had similar ADG and ADFI, but feeding the 5.9% EE cDDGS source reduced G:F compared with pigs fed the same inclusion rate of the higher oil cDDGS sources. Similarly, results of other studies have shown that reduced oil cDDGS can be included by up to 30% in pig diets without detrimental effects on growth performance when accurate ME or net energy (NE) and digestible AA values for cDDGS were used in diet formulations (Li et al., 2012; Kerr et al., 2015).

The high concentration of nonstarch polysaccharides (NSP; 25%) in cDDGS is one of the primary factors that reduce the ME relative to the gross energy content of cDDGS in pig diets (Kerr and Shurson, 2013; Jaworski et al., 2015). High NSP in cDDGS can be managed by using the NE system when formulating swine diets, but the results have been inconsistent. Gutierrez et al. (2014) reported that formulating high fiber cDDGS on a NE basis did not change growth performance of growing–finishing pigs compared with feeding cDDGS diets formulated on a ME basis, whereas Wu et al. (2016b) reported a decrease in ADG and G:F when pigs were fed diets containing cDDGS with lower NE content.

Supplementation of cDDGS diets with exogenous enzymes to improve fiber digestibility and energy has been evaluated in numerous studies during the past 20 years (Ndou et al., 2015; Swiatkiewicz et al., 2016; Moran et al., 2016). However, the growth performance and digestibility responses from adding exogenous feed enzymes to pig diets containing cDDGS have been inconsistent due to many factors such as the type(s) of enzyme included in the diets, amount of dietary substrates, ME and digestible amino acid (AA) of the basal diet, age of pig, and feeding duration (Adeola and Cowieson, 2011; Swiatkiewicz et al., 2016; Tsai et al., 2017). Although it is important to properly match the target substrates (i.e., nonstarch polysaccharides, proteins, and phytate) with using the appropriate enzymes, no meta-analysis summaries have been conducted and published to evaluate enzyme responses to diets containing cDDGS.

A systematic review involves the use of transparent and repeatable analytical methods of all relevant research to estimate mean effects (Sargeant et al., 2006). The use of a meta-analysis allows the results from multiple, independent studies identified, and critically evaluated in a systematic review, to be combined in homogeneous pools to address a more precise overall estimate of mean effects (Borenstein et al., 2009).

Therefore, the first objective of this systematic review and meta-analysis was to determine the effects of the dietary energy system (NE or ME), oil content of cDDGS, diet inclusion levels, and pig age on growth performance of pigs fed cDDGS diets. The second objective was to examine the efficacy of adding different types of exogenous enzymes, without or with phytase, to corn–SBM-based diets and cDDGS diets.

## MATERIALS AND METHODS

### Data Sources and Description of the Database

**Figure 1.**
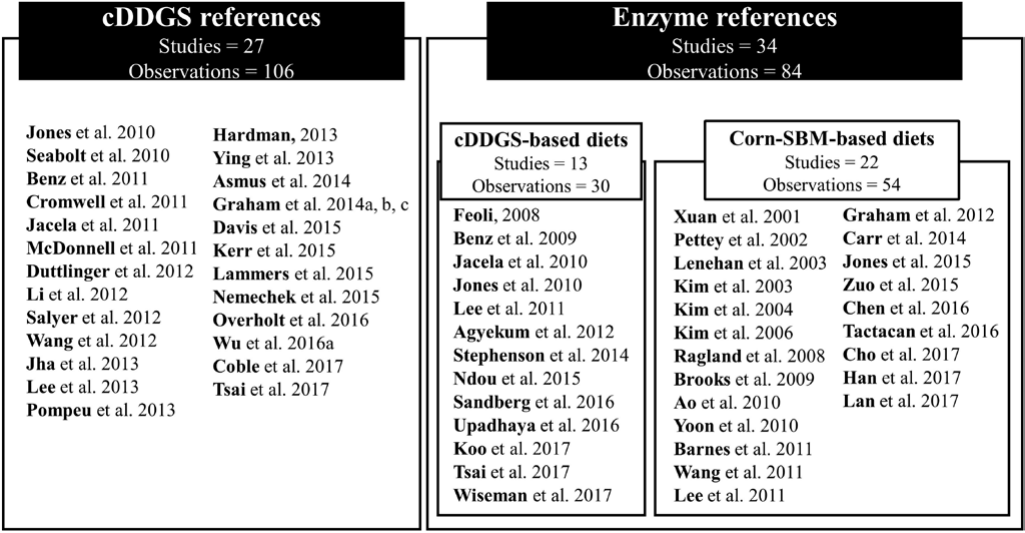
Number of studies and observations selected for the meta-analysis.

A literature search was conducted using the following electronic databases: PubMed (www.ncbi.nlm.nih.gov/pubmed), ISI Web of Science (www.webofknowledge.com), and Scopus (http://www-scopus-com). For the cDDGS diet dataset, the keywords used in the search included growth performance, distillers dried grains with solubles, pigs, piglets, and swine. The main criteria used for data selection included: 1) papers published from 2010 to 2018; 2) in vivo swine studies including corn–SBM-based diets as a control diet; 3) experimental diets were formulated using either ME or NE content and met the requirements of swine when comprised of corn–SBM and corn–SBM–cDDGS; 4) growth performance data (ADG, ADFI, and G:F) were reported; and 5) replicates (*n*) and variances (standard deviation, SD or standard error of mean, SEM), age of pigs, and duration of the study were provided. When feed:gain or feed conversion ratio data were reported, values were converted to G:F for consistency so that comparisons could be made between experiments. The final database used for determining the effects of adding cDDGS to corn–SBM-based based diets for swine included 106 observations from 26 peer-reviewed publications and one MS thesis, which were published between 2010 and 2018 (Figure 1).

For exogenous enzyme dataset, the keywords used in the literature search included carbohydrase(s), xylanase, mannanase, protease, glucanase(s), enzyme complex, enzyme cocktails, multicarbohydrases, pigs, swine, piglets, and growth performance. The main criteria used for data selection were: 1) papers published from 2001 to 2018; 2) in vivo swine studies including basal diet and basal diet plus exogenous enzyme; 3) basal diets were comprised of corn–SBM-based without any other high fiber ingredients and without or with cDDGS; 4) growth performance data were reported; and 5) replicates (n) and variances (SD or SEM) were provided. The final database used in the exogenous enzyme dataset included 84 observations from 27 peer-reviewed publication, one abstract, one PhD thesis, and five annual university swine day reports that were published between 2001 and 2018 (Figure 1).

### Statistical Analyses

To determine the effects of cDDGS inclusion rate in swine diets, mean difference was calculated by subtracting the mean ADG, ADFI, and G:F of pigs fed corn–SBM-based diet and cDDGS based diet and expressed as percentage (%) of pigs fed corn–SBM-based diet:


$$\begin{array}{ll} & Mean\;difference\;\\ & =\;\left(cDDGS\;\;corn-SBM\right)\;/\;corn-SBM\;\times \;100. \end{array}$$

Similarly, the effects of exogenous enzyme supplementation in pig diets containing corn–SBM-based or cDDGS, mean difference was calculated by subtracting the mean ADG, ADFI and G:F of pigs diets without or with enzyme and expressed as percentage (%) of pigs fed the control diet:


$$\begin{array}{ll} & Mean\;difference\;\\ & =\;\left(enzyme\;\;control\right)\;/\;control\;\times 100. \end{array}$$

The pooled standard error (SE) was expressed as the percentage corresponding to control.

The mean difference of publication biases was assessed using Egger’s regression test for funnel plot asymmetry (Egger et al., 1997). This test uses the Y-intercept = 0 from a linear regression of a normalized effect estimate (estimate divided by its standard error) with precision (reciprocal of the standard error of the estimate). There was no significant Y-intercept observed in the current study. The heterogeneity was quantified using the inconsistency index (*I*^2^-statistic), which was obtained by using the method-of-moments in SAS (Thompson and Higgins, 2002; Madden and Paul, 2011) and calculated as:


$$\begin{array}{l} I^{2}=\;100\%\;\times \;\left(Q-\;df\right)\;/Q \end{array}$$

where *Q* is Cochran’s heterogeneity statistic and df = the degrees of freedom. A separate meta-analysis was performed according to the Hedges–Olkin random-effects model using the means macro in SAS (Hedges and Vevea, 1998). A random-effects meta-analysis was used to account for the heterogeneity of the magnitude of response which was quantified by between-study variance (Huedo-Medina et al., 2006).

**Figure 2.**
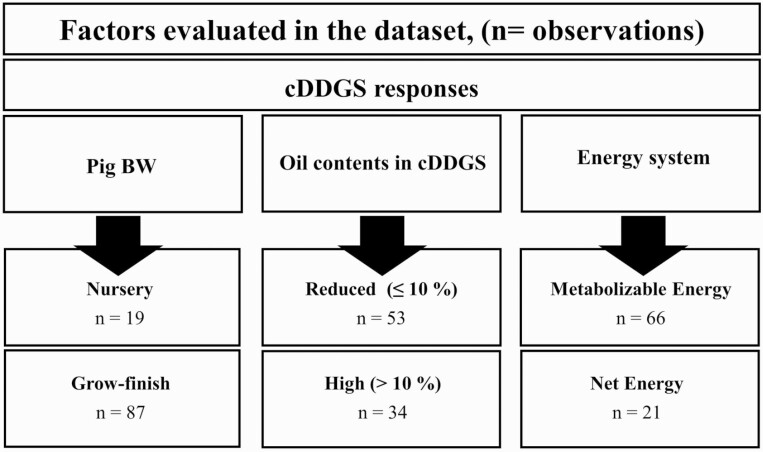
A subanalysis factors and number of observations selected in the corn distiller’s dried grains with solubles (cDDGS) response.

Data were also analyzed using PROC GLIMMIX (SAS Institute, Cary, NC) with individual observations serving as the statistical unit. The influence of cDDGS diet inclusion rate, oil content of cDDGS (reduced vs. high oil), age of pigs (nursery vs. growing–finishing), and energy system (ME vs. NE) were considered as fixed effects (Figure 2). Dietary cDDGS inclusion levels served covariates and individual studies were considered as random effects.

**Figure 3.**
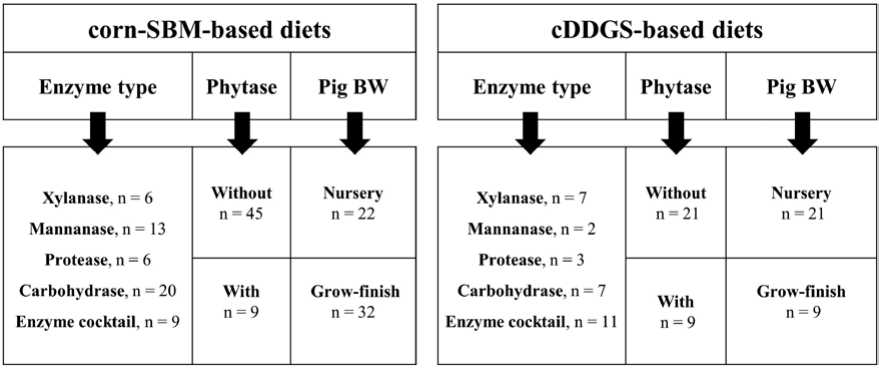
A subanalysis factors and number of observations (*n*) selected in the enzyme response in corn–soybean meal (SBM)-based and corn distiller’s dried grains with solubles (cDDGS)-based diets.

To determine the effects of exogenous enzyme supplementation in corn–SBM-based or cDDGS diets, the fixed effects included different single enzymes, combinations of enzymes, phytase presence (without or with for both the control and enzyme treatment), and age of pigs (nursery vs. growing–finishing). The enzyme types were categorized as single [xylanase (Xyl); mannanase (Man); protease (Pro)], and multi [carbohydrase (Carb) = combinations of carbohydrases; cocktail (Cock) = mixture of protease and single carbohydrase or protease and carbohydrase complex]. Figure 3 shows the subanalysis factors and number of observations selected for each enzyme response. The within-study sampling variances were predetermined by forcing the residual to be ‘1’ for all studies and to simultaneously fix the WEIGHT (which was a within-study weight) as the inverse of sampling error (Kaplan and Ferguson, 1999).

The estimated intercept (i.e., 1) and residue of each observation from the multivariable models were determined as an output variable to understand if the included factors in multivariable models contributed to publication bias and heterogeneity of effect size of the mean difference. All macro SAS codes previously described were based on the study by Madden and Paul (2009).

## RESULTS AND DISCUSSION

### General Evaluation of Growth Performance Responses of Feeding DDGS Diets

Among the 102 growth performance observations used in this meta-analysis, the majority (>65%) showed no changes in ADG, ADFI, and G:F when a portion of corn, soybean meal, and inorganic phosphorus was replaced with cDDGS (Table 1). However, approximately 27 % of the results reported in published studies showed a reduction in ADG, ADFI and G:F in pigs fed cDDGS-based diets compared with those fed corn–SBM-based-based diets. In general, there was a significant (*P* < 0.05) small percentage reduction in ADG (–1.86%), ADFI (–1.04%), and G:F (–1.18%) of pigs fed cDDGS diets compared with those fed corn–SBM-based diets (Table 2). However, the heterogeneity among studies was considerable, as indicated by *I*^2^ (> 67%) for ADG, ADFI, and G:F. A meta-analysis is not only important for pooling data from studies to increase the power of estimating the magnitude of treatment effects, but it is also crucial for investigating potential factors affecting heterogeneity (Thompson, 1994) because it is useful for identifying the factors or conditions (i.e., age of pigs, oil content of cDDGS, energy system used in diet formulation) that may benefit more from cDDGS inclusion. Therefore, subanalysis using the fixed effects of pig age, dietary inclusion rates of cDDGS, type of energy system used in diet formulation, and oil content of cDDGS was conducted to explore heterogeneity that may affect the outcome of adding cDDGS to corn–SBM-based diets.

**Table 1.** Effects of including corn distillers dried grains with solubles (cDDGS) in diets fed to nursery and growing–finishing pigs

			Responses to dietary corn DDGS^*a*^		
Item, %	Studies^*b*^	Observations	Increased	Reduced	Not changed
ADG	27	106	3	29	74
ADFI	27	106	12	28	66
G:F	27	106	11	28	67

^*a*^The number of significant and non-significant results.

^*b*^The studies selected in the dataset: Jones et al. (2010); Seabolt et al. (2010); Benz et al. (2011); Cromwell et al. (2011); Jacela et al. (2011); McDonnell et al. (2011); Duttlinger et al. (2012); Li et al. (2012); Salyer et al. (2012); Wang et al. (2012); Jha et al. (2013); Lee et al. (2013); Pompeu et al. (2013); Hardman, 2013; Ying et al. (2013); Asmus et al. (2014); Graham et al. (2014a, 2014b, 2014c); Davis et al. (2015); Kerr et al. (2015); Lammers et al. (2015); Nemechek et al. (2015); Overholt et al. (2016); Wu et al. (2016a); Coble et al. (2017); Tsai et al. (2017).

**Table 2.** The relative growth performance responses from feeding corn distillers dried grains with solubles (cDDGS) diets to nursery and growing–finishing pigs^*a*^

Item, %	Studies	Observations	*I*^2^, %^*b*^	Mean difference^*c*^	SE^*d*^	*P* value
All data						
ADG	27	106	74.7	–1.86	0.32	<0.01
ADFI	27	106	85.9	–1.04	0.33	<0.01
G:F	27	106	66.6	–1.18	0.31	<0.01
Nursery pigs, BW < 25 kg						
ADG	4	19	0.0	–4.27	0.94	<0.01
ADFI	4	19	37.8	–0.25	0.84	0.77
G:F	4	19	0.0	–1.99	0.61	<0.01
Growing–finishing pigs, BW ≥ 25 kg						
ADG	24	87	77.4	–1.68	0.31	<0.01
ADFI	24	87	87.4	–1.06	0.35	<0.01
G:F	24	87	70.7	–1.06	0.34	<0.01

^*a*^Data are presented as % relative differences which were calculated as (DDGS diet response – corn–soybean control diet response)/corn–soybean control diet response × 100). The standard error of each trial was converted to a pooled standard error divided by control value.

^*b*^*I*^2^ = the percentage of variation across studies that is due to heterogeneity rather than treatment changes.

^*c*^Mean difference = differences between DDGS diet and corn–soybean control diet expressed as a relative percentage.

^*d*^Standard error.

### Factors Affecting cDDGS Responses

#### Body weight.

 A subgroup meta-analysis was conducted to evaluate the effects of pig BW on the growth performance responses of pigs fed cDDGS-based diets (Table 2). The *I*^2^ values indicated that the percentage variability across studies was due to differences among studies and not due to random chance. In general, the greater the *I*^2^ value, the greater the likelihood that differences were not due to random chance, where a 25% (*I*^2^ = 25) is considered low chance, 50% (*I*^2^= 50) is considered medium chance, and 75% (*I*^2^ = 75) is considered the high likelihood that differences were not due to chance (Higgins and Thompson, 2002). For nursery pigs (BW < 25 kg), there were 3 studies with 15 observations that indicated dietary cDDGS inclusion decreased ADG (–4.27%) and G:F (–1.99%). However, low heterogeneity (*I*^2^ = 0%) suggest that the differences among studies can be explained by random factors and not by chance. For growing–finishing pigs, the heterogeneity (*I*^2^ > 71%) was considerable for ADG, ADFI, and G:F, suggesting that other factors such as dietary cDDGS inclusion rate, energy system used to formulate diets, and oil content of cDDGS likely contributed to the variance among studies.

#### Oil content of cDDGS.

A multivariable mixed model was applied to evaluate the effects of dietary cDDGS inclusion rates on growth performance responses of growing–finishing pigs as part of the evaluation of factors associated with the reduction in growth performance of pigs fed cDDGS-based diets compared with those fed corn–SBM-based-based diets (Table 3). Covariance analysis indicated that every percentage unit (%) increase in the inclusion level of cDDGS in growing–finishing pig diets was associated with a decrease (*P* < 0.01) in ADG (–0.10%) and ADFI (–0.09%), respectively. The oil content in cDDGS and type of energy system (ME or NE) used in diet formulation had no effect on ADG responses when pigs were fed cDDGS diets. However, the magnitude reduction was greater (*P* < 0.01) for ADFI, and less (*P* < 0.02) for G:F for pigs fed high-oil (≥10% EE) cDDGS sources compared with that fed reduced-oil (<10% fat) cDDGS sources. When diets included 25% cDDGS, pig fed high-oil cDDGS diets had a reduction in ADG (–2.38%, *P* < 0.05), and ADFI (–2.96%, *P* < 0.05), whereas pigs fed reduced-oil cDDGS diets had slightly less magnitude of reduction in ADG (–1.76%, *P* < 0.05), and G:F (–1.56%, *P* < 0.05).

**Table 3.** Mean differences in ADG, ADFI, and G:F between growing–finishing pigs (final body weight > 25 kg) fed diets without or with corn distillers dried grains with solubles (cDDGS) based on oil content of cDDGS sources (≥10% or <10%) and formulating diets on a ME or NE systems using a multivariable mixed model^*a*,*b*^

	Oil content			Energy system^*d*^			*P* value	
Item	High (≥10 %)	Reduced (<10%)	SE^*c*^	ME	NE	SE^*c*^	Oil	Energy
No. studies	9	16		19	5			
No. observations	34	53		66	21			
ADG	–2.38^**^	–1.76^**^	0.63	–1.69^**^	–2.45^**^	1.07	0.33	0.49
ADFI	–2.96^**^	–0.29	0.79	–1.51	–1.72	1.92	<0.01	0.92
G:F	0.24	–1.56^**^	0.73	–1.14^*^	–0.19	1.22	0.02	0.45

^**^Least square means differ from 0 (*P* < 0.05).

^*^Least square means differ from 0 (*P* < 0.10).

^*a*^The least square means the value at dietary DDGS level = 25 % were presented in the table. Studies that reported nursery pig data (final BW < 25 kg) were not included in the mixed model because all of these publications used the ME system for formulating diets and used only reduced oil DDGS sources.

^*b*^Covariance analysis indicated that every percentage unit increase in the inclusion level of cDDGS in growing–finishing pig diet was associated with a decrease (*P* < 0.01) in ADG (–0.10 %) and ADFI (–0.09 %), respectively.

^*c*^Standard error.

^*d*^Studies reported using either the metabolized energy (ME) or net energy (NE) system to formulate diets. A study by Li et al. (2012) used DE system for the diet formulation, thus excluded in the dataset.

Feed intake is often reduced when supplemental fats and oils are added to swine diets (Davis et al., 2015; Li and Patience, 2016). Therefore, high oil cDDGS sources increase the EE content of the diet which may result in decreased ADFI compared with feeding diets containing reduced-oil DDGS sources. The lack of change in G:F in pigs fed high oil cDDGS sources may be explained by the lack of difference in ADG and ADFI. The nonstarch constituents (i.e., protein, lipids, fiber, minerals, and vitamins) in cDDGS increase by approximately 3 times after starch is converted to ethanol during the dry-grind ethanol production process compared with the original concentrations in corn (Liu, 2011). However, the nutrient composition is variable among cDDGS sources, especially for EE content (Pedersen et al., 2014; Smith et al., 2015; Zeng et al., 2017), because the majority of the U.S. ethanol plants are partially extracting variable amounts of corn oil from the solubles prior to manufacturing cDDGS (U.S. Grains Council, 2018). The authors of the NRC (2012) published estimates of ME, NE, and digestible AA by separating cDDGS into 3 categories based on oil concentration (high-oil = > 10% EE, medium-oil, 6% to 9% EE, and low-oil, <4% EE) because they assumed that a reduction in oil concentration would be associated with a reduction in ME. However, there were limited published ME values for the concentration of medium-oil (6% to 9%; 3,801 kcal/kg ME) and low-oil (<4%; 3,476 kcal/kg ME) cDDGS at the time of publication. Since the publication of NRC (2012), subsequent studies have shown that the accuracy and usefulness of this designation are questionable because EE of cDDGS is a poor single predictor of ME for swine (Kerr et al., 2013; Urriola et al., 2014). Furthermore, the lower magnitude of reduced ADG in growing–finishing pigs fed reduced-oil cDDGS compared with that of pigs fed high-oil cDDGS suggests that EE of cDDGS is poorly correlated with ME. Therefore, a recommended (Urriola et al., 2014) and validated (Wu et al., 2016a) prediction equation based on cDDGS chemical composition should be used for more precise and accurate ME estimates compared with NRC (2012) values when formulating diet with reduced-oil cDDGS.

#### Energy system.

 When diets included 25% cDDGS, the use of ME system tended (*P* = 0.09) to slightly decrease G:F (–1.14%) in pigs fed cDDGS diets, whereas G:F (–0.19%) did not change in pigs fed cDDGS diets formulated on a NE basis. However, caution is needed when interpreting these results. First, the data set was unbalanced because it was comprised of 19 studies that used the ME system, but only five studies that used the NE system in diet formulation. Therefore, a larger data set of G:F responses for studies using the ME system versus the NE system may be a simple explanation for the differences observed. Second, the ME and NE values for cDDGS used in formulating diets varied considerably among these studies, and may not have been accurate for the specific cDDGS sources used. For example, Kerr et al. (2013) reported that ME content of 15 cDDGS sources with variable EE content ranged from 3,266 kcal/kg DM to 3,696 kcal/kg DM. This difference of 430 kcal/kg DM is likely great enough to affect the energy density of diets containing 25% cDDGS, and consequently, feed intake. Furthermore, most of NE values for cDDGS used in the studies evaluated were obtained from static reference values (Sauvant et al., 2004; NRC, 2012; Kerr et al., 2015) and may not have represented the actual NE content of the cDDGS source fed. Wu et al. (2016a) evaluated the accuracy of using static NE values as well as those derived from prediction equations and reported the inaccuracies that can occur when determining, which NE value to use for cDDGS when formulating diets (Wu et al., 2016a). Third, the ME dataset consisted of 12 studies where diets were formulated to be nonisocaloric diets, whereas seven studies evaluated diets formulated to be isocaloric using supplemental lipid sources. Because feed intake is dependent on the energy density of swine diets (Henry, 1985), the ME or NE contributions from other ingredients, such as supplemental lipids, and the assumed formulation values for ME or NE assigned to those ingredients, can also influence G:F responses from feeding cDDGS diets. However, despite these possible interpretations, our multivariable mixed model analysis might suggest that improved G:F can be achieved by using the NE system compared with the ME system when formulating diets containing high protein and fiber ingredients like cDDGS because the ME system overestimates the NE content due to increased heat increment (Noblet et al., 1994; Black, 1995; Kil et al., 2013). The advantage of using the NE system is greater when formulating diets containing more fibrous ingredients such as cDDGS, compared with corn–SBM-based diets (Le Bellego et al., 2001; Patience and Beaulieu, 2005).

#### Diet inclusion level of cDDGS.

There is increasing evidence that high (greater than 30%) dietary inclusion rates of cDDGS may negatively affect growth performance of pigs due to 1) inadequate SID threonine (Thr) to lysine (Lys), 2) excess SID leucine (Leu) interfering with SID isoleucine (Ile) and valine (Val), and 3) inadequate SID tryptophan (Trp) relative to Lys in the nursery and growing–finishing pigs diets. Corn DDGS contains a relatively high amount of total dietary fiber (31.4%; NRC, 2012), and high fiber diets increase secretion of mucin in the gastrointestinal tract (GIT) of pigs. Mucin is rich in Thr (constituting 28% to 35% of the total AAs, Mantle and Allen, 1981; Lien et al., 1997), and therefore, dietary fiber-induced intestinal mucin secretion may represent a significant endogenous loss of Thr (Nichols and Bertolo, 2008). In fact, an increase in the SID Thr to Lys ratio should be used when formulating swine diets containing increasing inclusion levels of dietary fiber (Mathai et al., 2016; Yang et al., 2019).

Corn DDGS contains relatively high concentrations of Leu relative to Ile and Val. These branched-chain AA (BCAA) are structurally similar and share the enzymes (branched-chain aminotransferase; BCAT) in the first steps of their catabolic pathway through the α-keto-acid dehydrogenase (BCKD) complex (Harris et al., 2004). Therefore, excess of one BCAA, particularly Leu, may result in increased catabolism of all three BCAA. Although this meta-analysis did not evaluate growth performance responses from dietary BCAA content at increasing diet inclusion rates of cDDGS, several studies have shown that the excess dietary SID Leu content in diets with high inclusion rates of cDDGS reduces ADFI and ADG in pigs (Rojo et al., 2016; Yang et al., 2019). Although current meta-analysis did not include an AA digestibility dataset due to lack of observations and considerable variance, the covariance analysis supports these associations and suggest that the increase in negative responses as dietary cDDGS levels increased may be partially due to excess Leu relative to Ile and Val.

 In addition, dietary excess of large neutral AA (LNAA = BCAA + Tyr, and Phe) in cDDGS may also cause depression of feed intake in pigs. The LNAA and Trp use the same nonspecific L-type AA transport crossing the blood-brain barrier (Fernstrom and Wurtman, 1971; Meunier-Slalün et al., 1991). Therefore, excess LNAA may limit Trp for transport across the barrier and reduce serotonin production in the hypothalamus because serotonin is synthesized from the dietary Trp (Shen et al., 2012). The consumption of high levels of cDDGS in the diet may decrease diet Trp:LNAA, reducing the Trp availability by the increased competition with LNAA to cross the blood-brain barrier (Lyons and Truswell, 1988; Kerr et al., 2002). Thus, formulating diets containing high inclusion rates of cDDGS should increase SID Trp:Lys to prevent reductions in feed intake.

### General Evaluation of Exogenous Enzyme Efficacy in DDGS and Corn–SBM-based Diets

Many published studies reported no change in ADFI (48 of 54 for corn–SBM-based diets and 28 of 30 for cDDGS diets) when feeding pigs without or with exogenous enzymes (Table 4). Improvements in ADG and G:F were observed in 31% and 37% of observations, respectively, when exogenous enzymes were supplemented in CSB diets, whereas exogenous enzyme supplementation improved ADG in only 10% of observations and G:F in 23% of observations when exogenous enzymes were supplemented in cDDGS diets.

**Table 4.** Effects of dietary inclusion of feed enzymes on growth performance of nursery and growing–finishing pigs fed corn–soybean meal (SBM)- or corn distillers dried grains with solubles (cDDGS)-based diets

			Responses to feed enzyme inclusion^*a*^		
	Studies	Observations	Increased	Reduced	Not changed
Corn–SBM-based diets^*b*^					
ADG	22	54	17	1	36
ADFI	22	54	3	3	48
G:F	22	54	20	0	34
cDDGS-based diets^*c*^					
ADG	13	30	3	1	26
ADFI	13	30	2	0	28
G:F	13	30	7	1	22

^*a*^The number of significant and nonsignificant results.

^*b*^The studies that evaluated enzyme addition to corn–SBM-based diets included: Xuan et al. (2001); Pettey et al. (2002); Kim et al. (2003); Lenehan et al. (2003); Kim et al. (2004); Kim et al. (2006); Ragland et al. (2008); Brooks et al. (2009); Ao et al. (2010); Yoon et al. (2010); Barnes et al. (2011); Lee et al. (2011); Wang et al. (2011); Graham et al. (2012); Carr et al. (2014); Jones et al. (2015); Zuo et al. (2015); Chen et al. (2016); Tactacan et al. (2016); Cho et al. (2017); Han et al. (2017); Lan et al. (2017).

^*c*^The studies that evaluated enzyme addition to cDDGS diets included: Feoli (2008); Benz et al. (2009); Jacela et al. (2010); Jones et al. (2010); Lee et al. (2011); Agyekum et al. (2012); Stephenson et al. (2014); Ndou et al. (2015); Sandberg et al. (2016); Upadhaya et al. (2016); Koo et al. (2017); Tsai et al. (2017); Wiseman et al. (2017).

Supplementing exogenous enzymes in corn–SBM-based diets increased (*P* < 0.01) the mean difference of ADG and G:F by 1.94% and 2.65%, respectively (Table 5). However, the heterogeneity of ADG and G:F was considerable (*I*^2^ > 88%). There were 13 studies with 30 observations using corn–SBM-based diets containing cDDGS. Dietary exogenous enzyme addition increased (*P* < 0.01) the mean difference of ADG (1.94%) and G:F (2.65%) of pigs fed corn–SBM-based-based diets, whereas pigs fed cDDGS-based diets had increased (*P* < 0.01) mean difference for ADG (2.67%) and G:F (1.87%). However, observations from feeding both corn–SBM-based- and cDDGS-based diets resulted in considerable heterogeneity (*I*^2^ =83.1% in corn–SBM- and 63.8% in cDDGS-based diets, respectively). Therefore, we explored factors (such as types of enzyme, fiber source, or level added to basal diets, age of pigs, and feeding duration) affecting the variability among studies.

**Table 5.** Effects of including feed enzymes on the mean difference (relative improvement) on growth performance of pigs fed diets containing corn–soybean meal or corn distillers dried grains with solubles (cDDGS) diets^*a*^

Item, %	Studies	Observations	*I*^2^, %^*b*^	MD^*c*^, %	SE^*d*^	*P* value
Corn–SBM-based diets						
ADG	22	54	88.8	1.94	0.64	<0.01
ADFI	22	54	69.5	–0.63	0.40	0.12
G:F	22	54	91.0	2.65	0.54	<0.01
cDDGS-based diets						
ADG	13	30	60.0	2.67	0.54	<0.01
ADFI	13	30	62.5	–0.19	0.56	0.74
G:F	13	30	68.8	1.87	0.62	<0.01

^*a*^Improvement = (enzyme – control)/control × 100%; the SE of trials was converted to SE/control value.

^*b*^Describes the percentage of variation across studies that is derived from heterogeneity rather than chance. High heterogeneity (*I*^2^ > 70 %) indicates very high between-study variance.

^*c*^Mean difference (percentage of improvement).

^*d*^Standard error.

### Factors Affecting Enzyme Responses in cDDGS and Corn–SBM-based Diets

A multivariable mixed model was applied to evaluate the effects of dietary enzyme supplementation on the percentage of improvement in growth performance of pigs fed corn–SBM- or cDDGS-based diets (Table 6). For corn–SBM-based diets, the type of enzyme increased (*P* < 0.05) the mean difference for ADG, and tended (*P* = 0.10) to increase the mean difference for G:F. Diets supplemented with protease (7.29%) had a greater (*P* < 0.05) mean difference for ADG compared with those supplemented with mannanase (1.50%) or carbohydrases (0.97%). However, no enzyme response was observed in cDDGS-based diets. Age of pigs and the addition of phytase in combination with carbohydrases or proteases did not affect exogenous enzyme efficacy on growth performance when pigs were fed either corn–SBM- or cDDGS-based diets. The heat treatment during the production of cDDGS can affect the digestibility of protein (Meade et al., 2005) because of high drying temperatures, with an inlet temperature as high as 500°C and an outlet temperature of 150 °C (Bhadra et al., 2011). These high temperatures used drying process can cause Maillard reactions resulting in reduced amino acid digestibility (Pahm et al., 2008; Almeida et al., 2013).

**Table 6.** Effects of dietary enzyme supplementation on improvement (%) of growth performance of pigs fed corn–soybean meal- or with corn distillers dried grains with solubles (cDDGS)-based diets from multivariable mixed models^*a*^

	Enzyme type^*b*^														
	Single			Multi			Phytase^*d*^			Pig BW^*e*^			*P* value^*f*^		
Item, %	Xyl	Man	Pro	Car	Cock	SE^*c*^	–	+	SE	Nursery	G-F	SE	ENZ	PHY	BW
Corn–SBM-based diets															
Studies	4	6	2	8	3		16	6		10	13				
Observations	6	13	6	20	9		45	9		22	32				
ADG	2.48^abc^	1.50^bc^	7.29^**a^	0.97^c^	2.64^*ab^	1.53	3.31^**^	2.64	1.12	3.00^**^	2.96^**^	1.02	0.05	0.68	0.98
ADFI	–0.53	–0.50	0.51	–0.93	1.53	1.14	–0.44	0.47	0.84	–0.48	0.51	0.79	0.12	0.46	0.34
G:F	3.10	1.83	6.84^**^	2.17	1.65	1.64	3.55^**^	2.64	1.30	3.67^**^	2.52^*^	1.20	0.10	0.66	0.47
cDDGS-based diets															
Studies	3	1	2	3	5		9	4		8	6				
Observations	7	2	3	7	11		21	9		21	9				
ADG	1.64	3.32^**^	2.33	2.54	3.20^**^	1.26	2.26^**^	2.95^**^	0.84	2.51	2.70^**^	1.01	0.70	0.36	0.91
ADFI	–0.48	–0.42	1.77	1.25	0.45	1.27	0.52	0.51	0.87	0.68	0.36	1.01	0.60	0.99	0.84
G:F	3.01^**^	2.82^**^	1.35	0.97	2.82^**^	1.17	2.12^**^	2.26^**^	0.76	2.62^*^	1.77^*^	0.83	0.73	0.86	0.44

^a^– ^c^Least square means with a different superscript are significantly different (*P* < 0.05).

^**^Mean values differ from 0 (*P* < 0.01).

^*^Mean values differ from 0 (*P* < 0.05).

^*a*^Improvement = (enzyme – control)/control × 100%.

^*b*^Different combination of enzymes: single (Xyl = xylanase, Man = mannanase, or Pro = protease), and multi (Car = mixture of carbohydrases; Cock = cocktail mixture of protease plus single or multiple carbohydrases).

^*c*^Standard error.

^*d*^Supplementing carbohydrases/protease in the presence (+) or absence (–) of phytase in the diet.

^*e*^Pig BW = nursery (nursery pigs, <25 kg BW) and G–F (growing–finishing pigs, >25 kg BW).

^*f*^Probability value of enzyme (ENZ), phytase (PHY), and pig BW (BW).

The efficacy of adding exogenous enzymes to swine diets requires matching appropriate enzymes to target substrates. The predominant noncellulosic polysaccharides in cDDGS are arabinoxylose (12.3% to 17.2%), which is mainly insoluble (Pedersen et al., 2014). Although only three studies evaluated the addition of proteases to corn–SBM-based diets in our meta-analysis, the magnitude of improvement (*P* < 0.01) was greater than responses from adding single carbohydrases, multiple carbohydrases, or a cocktail mixture of carbohydrases and protease (Table 6). Although no overall enzyme effect was observed, the addition of mannanase or cocktails of carbohydrases and protease improved (*P* < 0.01) ADG and G:F in cDDGS diets. The protein in SBM is comprised of approximately 40% glycinin and 30% β-conglycinin, which are considered to be anti-nutritional factors and are poorly tolerated in the digestive tract of young pigs (Maruyama et al., 1999). The glycinin damages the intestinal morphology by villus atrophy and crypt hyperplasia, leading to reduce protein and lipid metabolism in young pigs (Li et al., 1991; Zhao et al., 2010). Proteases have been added to nursery pig diets to reduce the antigenic challenge of soybean meal and improve growth performance of young pigs (Zuo et al., 2015; Pan et al., 2016; Park et al., 2020). Based on the current meta-analysis, it appears that the inclusion of protease in corn–SBM-based diets provided a greater benefit for improving ADG and G:F of pigs compare with other single or multi enzymes in corn–SBM-based diets. In contrast, although the number of studies and observations evaluating growth performance responses from adding protease to cDDGS diets were limited, protease addition had no effect on ADG or G:F. Corn DDGS has a more complex fiber-starch-protein matrix compared to corn (Jha et al., 2013).

In the enzyme response dataset used in this meta-analysis, 3 studies evaluated the use of Phyzyme (Danisco Animal Nutrition/Dupont, Marlborough, Wiltshire, UK), two studies provided no information on phytase source, and one study evaluated the use of Natuphos (BASF Corporation, Florham Park, NJ) in corn–SBM-based diets, whereas two studies evaluated the use of Optiphos (Enzyvia LLC, Sheridan, IN), two studies evaluated Axtra PHY and Phyzyme (Danisco Animal Nutrition/Dupont, Marlborough, Wiltshire, UK) in cDDGS diets. Unfortunately, most phytases included in the current meta-analysis dataset are microbial origin type 6-phytase (one type 3-phytase, seven type-6 phytases, and two unknown types of phytases), which limited our ability to conduct further analysis. Phytase effects can be specified based on the origin (bacterial or fungal) and type (3- or 6-) based on the source and the site initiating the dephosphorylation of phytate molecules at different positions on the inositol rings (Adeola and Cowieson, 2011). Exogenous phytase has been used in pig diets to eliminate the anti-nutritional effects of phytate (IP6) over the last five decades (Adeola and Cowieson, 2011). Although research on the use of phytase in combination with carbohydrases has suggested that there may be the synergistic effect on the growth responses of pigs, there is a lack of studies to evaluate the efficacy of these combined enzyme responses in cDDGS-based pig diets.

## CONCLUSION

The majority (>65%) of published observations showed no changes in ADG, ADFI, and G:F when a portion of corn, soybean meal, and inorganic phosphorus was replaced with cDDGS. However, when the addition of cDDGS to swine diets resulted in a reduction in ADG and G:F, the magnitude of reduction appeared to be greater for nursery pigs compared with growing–finishing pigs. Factors such as dietary cDDGS levels and oil content of cDDGS sources may influence the magnitude of reduction in ADG and G:F. Enzyme supplementation does not appear to improve growth performance in cDDGS-based diets, but the inclusion of protease or enzyme cocktail in corn–SBM-based-based pig diets improved ADG and G:F. Regardless of these overall responses, cDDGS continues to be an important and economically justifiable alternative energy and protein source which can be used effectively in swine diets in the global feed industry. However, variability in energy and digestible AA content among cDDGS sources continues to challenge nutritionists to use accurate ME, NE, and SID AA values in diet formulation in precision feeding programs.
